# Inflicting Significant Losses in Slaughtered Animals: Exposing the Hidden Effects of Parasitic Infections

**DOI:** 10.3390/pathogens12111291

**Published:** 2023-10-29

**Authors:** Hafiz Muhammad Rizwan, Hafiz Muhammad Zohaib, Muhammad Sohail Sajid, Haider Abbas, Muhammad Younus, Muhammad Umar Farid, Tahira Iftakhar, Hizqeel Ahmed Muzaffar, Syed Soban Hassan, Muhammad Kamran, Muhammad Hussnain Raza, Muhammad Haziq Bajwa

**Affiliations:** 1 Section of Parasitology, Department of Pathobiology, KBCMA College of Veterinary and Animal Science, Narowal, Sub Campus UVAS, Lahore 54000, Pakistan; hmzohaib456@gmail.com (H.M.Z.); haider.abbas@uvas.edu.pk (H.A.); 2 Department of Parasitology, Faculty of Veterinary Science, University of Agriculture, Faisalabad 38000, Pakistan; drsohailuaf@hotmail.com (M.S.S.); tahiraiftakhar1234@gmail.com (T.I.); 3 Section of Pathology, Department of Pathobiology, KBCMA College of Veterinary and Animal Science, Narowal, Sub Campus UVAS, Lahore 54000, Pakistan; younusrana@uvas.edu.pk; 4 Section of Meat Science, Department of Animal Sciences, KBCMA College of Veterinary and Animal Sciences, Narowal, Sub Campus UVAS, Lahore 54000, Pakistan; umar.farid@uvas.edu.pk; 5 Faculty of Veterinary Sciences, KBCMA College of Veterinary and Animal Sciences, Narowal, Sub Campus UVAS, Lahore 54000, Pakistan; hafizhizqeel32@gmail.com (H.A.M.); sobanh813@gmail.com (S.S.H.); kamran854515@gmail.com (M.K.); hussnainhr072@gmail.com (M.H.R.); haziqbajwa7@gmail.com (M.H.B.)

**Keywords:** ruminants, gastrointestinal parasites, prevalence, economic losses, burden, Pakistan

## Abstract

We started a campaign in the heart of Faisalabad, Punjab, Pakistan, to expose the hidden threats of parasitic illnesses in ruminants and the severe financial consequences associated with them. Our in-depth investigations focused on the prevalence, impact, and astounding financial losses brought on by organ contamination in slaughtered animals. Of the 384 slaughtered ruminants examined for gastrointestinal parasites, a prevalence of 44.79% was recorded. It is interesting to note that we found no conclusive association between parasitic infection and the various ruminant species under study (*p* > 0.05). However, goats (52.0%) had the highest numerical prevalence of parasitic infection, followed by cattle (46.1%), buffalo (46.0%), and sheep (34.7%) in that order. A significant finding (*p* < 0.05) showed that the majority of animals had light parasitism (46.5%), as opposed to those with moderate (30.2%) or severe loads (23.2%). Our research revealed substantial (*p* < 0.05) relationships between ruminant age, sex, and parasitic infection prevalence. In comparison to females (56.4%) and adults (48.1%), males (36.1%) and young (36.9%) ruminants showed considerably decreased infection rates (*p* < 0.05). On the other hand, we discovered a non-significant (*p* > 0.05) association between the months and the prevalence of parasitic infection. As a result of the condemnation of contaminated organs such as the rumen, lungs, and liver, an estimated financial loss of PKR 133,731,400 (USD = 466,939.2) was incurred. The yearly economic losses caused by liver condemnation were much greater than those caused by rumen and lung condemnation (*p* < 0.05). Our research not only reported a significantly higher abundance but also economic threats of the parasitic diseases among the slaughtered animals in Faisalabad, Punjab, Pakistan. Our findings highlighted the critical need for preventive and therapeutic interventions for parasitic infections in animals, in order to mitigate the economic losses through strengthened animal health.

## 1. Introduction

The condition known as gastrointestinal (GI) parasitism, which results in malnutrition, anaemia, diarrhoea, and stunted growth in ruminants, is brought on by parasites dwelling in their digestive tracts [1]. These GI parasites have a significant global impact that results in significant financial losses [2]. In the case of ruminants, GI parasites act as silent offenders, causing impaired weight gain, lowered production, digestive problems, compromised reproduction, condemnation of organs, and even mortality [3]. Beyond the clinical effects, financial losses attributable to parasitic infections include stunted growth and lowered meat production, decreased milk and wool output, raised mortality rates, especially youngstock, and increased input costs for parasite management strategies [4]. Hence, it is crucial to comprehend how common parasitic diseases affect ruminants in each given area.

Ruminants serve as a vital source of food and revenue for innumerable populations in underdeveloped nations, and they are essential to their well-being [5]. However, parasitic illnesses have the potential to endanger not just the health and welfare of these animals but also the overall financial stability of the agricultural ecosystem [6]. Mitigating the hidden costs of parasitic diseases becomes essential for maintaining both rural livelihoods and food security, given the reliance on agriculture by many regions in different parts of the world [7,8].

The incidence and general effects of parasite diseases on ruminants were well understood before the start of this study, but the subtleties of this problem in Faisalabad, Punjab, remained largely unexplored. During the study, we observed that in Faisalabad, access to veterinary care for ruminants is a critical aspect of livestock management (personal observation). However, the prevailing scenario suggests that many farmers tend to underutilize veterinary services for routine and mild infections, often seeking professional assistance only when confronted with severe health issues in their animals. For minor infections, these farmers often rely on their own knowledge and traditional remedies to address the problems. Unfortunately, this practice, although cost-effective in the short term, may not always provide effective solutions. Moreover, the management strategies employed by small-scale farmers in the region often fall short of optimal standards. This lack of adequate management practices, coupled with insufficient access to veterinary care, can have a substantial impact on the livelihoods of these farmers [9,10,11]. 

By providing a thorough evaluation of the prevalence, burden, and financial effects of parasitic diseases among ruminants in this area, our research intends to bridge this knowledge gap. The aim of the present study is to determine the prevalence and estimate the economic losses due to the condemnation of ruminant organs. By exposing the hidden aspects of this issue, we hope to improve our comprehension of the particular difficulties experienced by the livestock community in developing countries and, as a result, offer evidence-based suggestions to lessen the devastating costs brought on by parasitic illnesses, especially in the smallholder subsistence farming systems of resource-poor communities.

## 2. Materials and Methods

### 2.1. Study Area and Sample Size

The study was carried out in the district of Faisalabad, formerly known as Lyallpur. It is the second-largest metropolis in the eastern province of Punjab and the third-most populous city in Pakistan [12]. Faisalabad, Pakistan, had a range of climatic conditions from January to June 2023. From about 40% in January to about 60% in June, the average humidity varied. During this time, the average temperature increased gradually, going from about 15 °C in January to about 35 °C by June. It rained sporadically, with little precipitation being recorded, especially in the later months, signaling the start of the region’s hot and dry summer (data collected from the metrological department of Faisalabad).

The present study was conducted over a period of six months, from January 2023 to June 2023. According to the following formula given by Thrusfield et al. [13], 384 samples were collected from three selected slaughterhouses in the district of Faisalabad using stratified random sampling and proportional allocation at a 95% confidence level:

$$n=\frac{1.96^{2}P_{\exp}\;(1-P_{\exp})}{d^{2}}$$

where *n* = sample size; *P*_exp_ = expected prevalence; *d*^2^ = desired precision

For ruminants, a sample size of 384 was determined using an expected prevalence of 50% and a 5% level of precision. The district of Faisalabad has a total ruminant population of 2,864,889, according to data from the local veterinary facility. In the district of Faisalabad, ruminants are distributed as follows: goat = 744,918, cattle = 760,632, sheep = 711,753, and buffalo = 647,586 (data collected from the livestock department of Faisalabad). The following formula was used to determine the number of animals from each of the ruminant species (considered a stratum) in the district of Faisalabad that needed to be sampled:

$$n_{k}=n\frac{N_{k}}{N}$$

where *N* = population size; *N_k_* = population size of stratum; *n* = total sample size; *n_k_* = sample size from each stratum.

Sheep and goats were considered young in the current study if they were under 9 and 12 months old, respectively. When they were older than 9 months and 12 months, respectively, they were regarded as adults. Cattle and buffalo were categorized as young if they were under 24 months old and as adults if they were over 24 months. The age of the animals was determined via dental formula analysis and information provided by butchers and farmers.

### 2.2. Collection of Faecal Samples

Before slaughter, ruminant faeces (n = 384) were collected using the established procedures outlined by Soulsby [14]. Briefly, 3 parts formalin and 1 part sample were added to plastic bottles containing 10% formalin to hold 10 grams (g) of faeces per rectum. Age, sex, and species-specific labels were applied to plastic bottles before they were delivered to the Department of Parasitology at the University of Agriculture, Faisalabad (UAF), for additional processing in accordance with established procedures. Before being processed further, the samples were kept refrigerated (4 °C).

### 2.3. Examination of Different Organs

The entire gastrointestinal system and related visceral organs (rumen, abomasum, large and small intestine, liver, and lungs) were examined for parasitic infections and the collection of parasites. A systematic visual examination, palpation, and, if necessary, sharp incisions were made to the organs. The liver and lungs were examined for the presence of adult *Fasciola* and hydatid cysts, and the rumen was inspected for ruminal flukes, e.g., *Paramphistomum* spp. If any of these parasites were detected, they were collected on-site. The abomasa were examined by making a single incision, and they were checked for the presence of worms, e.g., *Haemonchus* or *Ostertagia*. Furthermore, the intestinal contents of each animal were collected in separate containers and transported to the Molecular Parasitology and One Health (MPOH) Lab at the University of Agriculture, Faisalabad (UAF), for further examination. The collected parasites were gently washed in water and preserved in 70% ethanol. The parasites were then grossly and microscopically classified according to Soulsby’s [14] description of their morphological characteristics.

### 2.4. Egg Identification and Quantitative Faecal Examination 

The standard sedimentation and flotation techniques (indirect coprological examination) were used to detect parasite eggs. Parasite species were identified based on the size and shape of their eggs. Quantitative assessment of parasite burden, i.e., egg per gram (EPG), was performed using the modified McMaster technique [15]. Briefly, 3 g of a thoroughly mixed faecal sample was obtained and homogenized in 42 mL water. Following homogenization, samples were sieved through a fine sieve (0.15 mm aperture), and the filtrate was collected in a centrifuge tube. Tubes were centrifuged at 1500 rpm for 2 min. The supernatants were discarded, and the volume was maintained by adding to or refilling the tubes with the saturated salt solution (NaCl). Following refilling, tubes were thoroughly mixed, and the solution was added into both chambers of a McMaster slide to count the number of eggs in each chamber. According to Rizwan et al. [16], a light infection was defined as 100 to 800 EPG, a moderate infection as 801 to 1200, and a high infection as more than 1200.

### 2.5. Determination of Economic Loss Due to Organs Condemnation

To calculate the economic losses, all organs presumably affected by the parasites were viewed as being condemned. The annual losses from organ condemnation were calculated using the total number of animals slaughtered in the abattoir annually and the average retail price of organs at the abattoir. The average market price of the organs was ascertained through conversations with butchers and slaughterhouse personnel using participatory approaches. The abattoir record had historical data from earlier years, which were used to compute the annual slaughter rate. The method described by Jaja et al. [17] was used to calculate the yearly financial loss caused by the condemnation of the organs, as given below:

Yearly financial loss = mean number of ruminants slaughtered per year × mean cost of organ × prevalence.

### 2.6. Statistical Analyses 

Information collected from the animal sources, including age, sex, and species, was recorded in a Microsoft Excel, 2010 spreadsheet and coded for subsequent analysis. The epidemiological data underwent analysis using Pearson’s Chi-square (χ^2^) test to determine significant associations within different groups. All tests for statistical significance were conducted at a threshold of *p* < 0.05, utilizing SPSS 17.0 (SPSS Inc., Chicago, IL, USA).

## 3. Results

In the Faisalabad district, the overall prevalence of parasitic infection was 44.8%. The association between parasitic infection and species of ruminants proved insignificant (*p* > 0.05). However, goats (52.0%) had the highest prevalence of parasitic infection, followed by cattle (46.1%), buffalo (46.0%), and sheep (34.7%) in that order. In ruminants, there was a substantial (*p* < 0.05) association between age (X^2^ value = 4.598) and sex (X^2^ value = 15.667) and the frequency of parasitic infection. Males and young ruminants showed a considerably lower prevalence than females and adult ruminants (*p* < 0.05). A connection between the prevalence of parasitic infection and the month of slaughter was not significant (*p* > 0.05). A light burden of parasitic infection was present in the majority of the ruminants, which was significantly (*p* < 0.05) higher than a moderate and high burden. The ruminant population of district Faisalabad was significantly (*p* < 0.05) more commonly infected with one parasitic species compared to >1. Table 1 presents the frequency distribution of parasitic infection in ruminants in the district of Faisalabad, Punjab, Pakistan.

**Figure 1:**
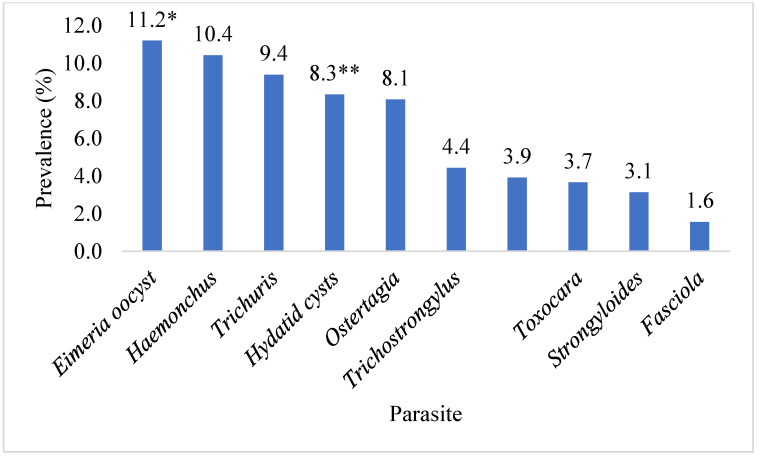
The prevalence of parasitic species identified in slaughtered ruminants in the abattoirs of Faisalabad, Punjab, Pakistan. * identified only through faecal examination; ** identified only through visual examination.

**Figure 2:**
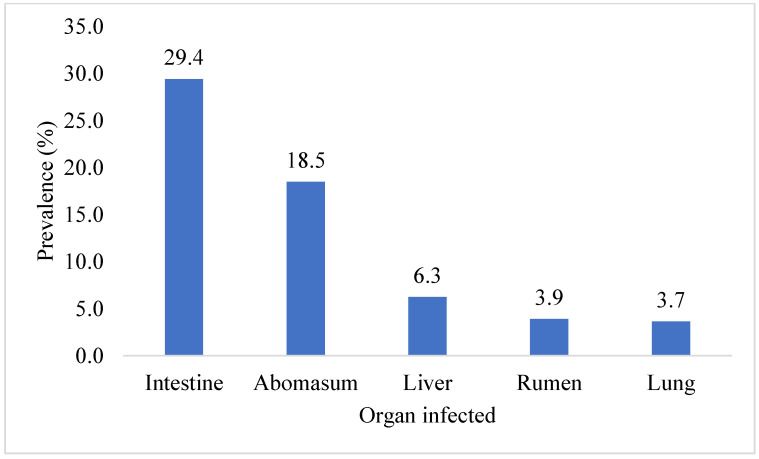
Overall prevalence of organs infected with parasites in Faisalabad.

**Figure 3:**
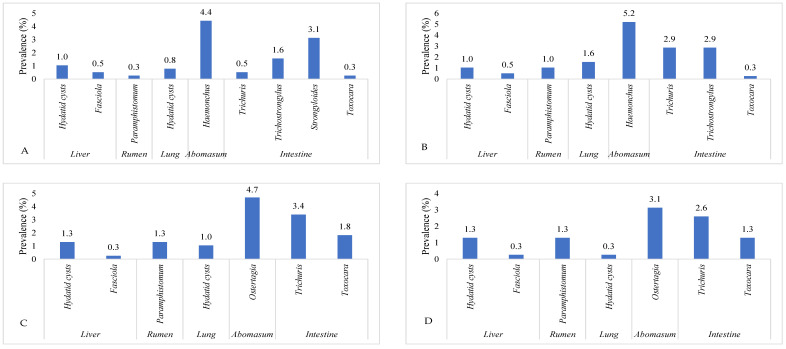
The prevalence of various parasitic species in different organs of different ruminant species in Faisalabad, Punjab Pakistan. (**A**) = sheep, (**B**) = goat, (**C**) = cattle, and (**D**) = Buffalo.

This investigation identified ten parasite species in total. The highest prevalence of *Eimeria* oocyst (11.2%) was found, followed by *Haemonchus* (10.4%), *Trichuris* (9.4%), Hydatid cysts (*Echinococcus*) (8.3%), *Ostertagia* (8.1%), *Trichostrongylus* (4.4%), *Paramphistomum* (3.9%), *Toxocara* (3.7%), *Strongyloides* (3.1%), and *Fasciola* (1.6%) species. Ruminants exhibited the presence of adult and egg stages of nine parasitic species, along with one intermediate form (hydatid cyst). The prevalence of *Eimeria* oocyst was significantly (*p* < 0.05) higher than that of other parasite species. Figure 1 shows the prevalence of parasite species found in the ruminant population of the Faisalabad district. A significantly (*p* < 0.05) higher prevalence of parasitic infection was detected in the intestines (29.4%), followed by the abomasa (18.5%), livers (6.3%), rumens (3.9%), and lungs (3.7%). The data on the organs of the slaughtered animals infected with parasitic infections in Faisalabad are given in Figure 2. The prevalence of hydatid cysts was higher in the liver (4.7%) than in the lung (3.7%). However, only *Fasciola* spp. was found in the liver (1.6%) of the ruminants. Across all categories of ruminants, only *Paramphistomum* spp. (3.9%) were found in the rumen. Small ruminants had *Haemonchus* spp. (10.4%) infections, while large ruminants exhibited *Ostertagia* spp. (8.1%) infections in their abomasa. In the intestines of ruminants, the identified adult parasites included *Trichuris* spp., *Trichostrongylus* spp., *Strongyloides* spp., and *Toxocara* spp. The prevalence of various parasitic species in different organs of the different slaughtered ruminants in the abattoirs of Faisalabad is presented in Figure 3.

Among the 172 infected ruminants, 112 (65.1%) were infected with a single parasite species. The most prevalent single parasitic infection in ruminants was *Eimeria* (15.1%), followed in order by *Trichuris* (13.9%), *Haemonchus* (12.8%), *Ostertagia* (11.6%), *Toxocara* (5.2%), *Strongyloides* (3.5%), and *Trichostrongylus* (2.9%). In the present study, twenty different kinds of pairs of parasite species were found in 46 (26.7%) ruminants. Twelve different combinations of three parasites were present in 14 (8.1%) ruminants. Parasites present as singles, pairs, and combinations of three are listed in Supplementary Table S1.

The total estimated annual economic losses due to the condemnation of organs (livers, lungs, and rumens) with GI parasites were PKR 133,731,400/- (USD = 466,939.2). The estimated annual economic losses due to liver condemnation (PKR = 95,828,080; USD = 334,595.3) were four times higher than those due to the rumen (PKR = 24,991,200; USD = 87,259.8) and seven times higher than those due to the lungs (PKR = 12,912,120; USD = 45,084.2/-). The estimated annual economic losses due to the condemnation of organs with parasitic infection are given in Table 2.

## 4. Discussion

Gastrointestinal parasitic infections pose significant challenges in ruminant production, particularly in tropical and subtropical regions. Moreover, these infections can lead to substantial economic losses by reducing weight gain, food intake, fertility rates, high cost of treatment, and mortality in severely parasitized animals. Additionally, the condemnation of various organs due to parasitic infection significantly contributes to financial losses [18].

Numerous studies have been conducted in Pakistan to determine the prevalence of GI parasites, with greater rates found in Cholistan (78.1%), Chakwal (63.33%), and Jatoi (52%), compared to the present study [19,20,21]. According to studies conducted by Rahman et al. [22] in Bangladesh, who reported a prevalence of 63.4%, and Dugassa et al. [23] in Ethiopia, who recorded a prevalence of 71.88%, these regions exhibited higher rates of gastrointestinal parasites compared to the rates observed in our current study. Conversely, Malathi et al. [24] documented lower prevalence rates (30.73%) of GI parasites in ruminants as compared to our study. Variation in prevalence can be linked to a number of variables, including local environmental circumstances, farming and animal husbandry techniques, the use of dewormers, and other factors affecting parasite transmission and persistence [7,25].

The significant role of parasitic infections as a major health concern in world ruminant populations is highlighted by the high incidence of parasites in the analyzed ruminant flocks [26,27]. Rahman et al. [22] noted increased infection rates in Pakistani sheep (60.67%) and goats (64.09%). In addition, Khan et al. [28] discovered that sheep (44.17%) had a considerably greater prevalence of GI helminths than goats, buffalo, and cattle (40.15%, 39.82%, and 33.68%, respectively). Another study by Raza et al. [3] reported slightly higher prevalence rates in cattle (51%), sheep (62%), and goats (52%), but comparable prevalence in buffalo (47%). In this study, compared to other ruminant species, goats had the highest prevalence of GI parasitic infections (52%), as compared to other species. This conclusion is in line with research done in Ethiopia, which reported a higher prevalence in goats (83%) compared to other species [29]. However, our results were not in line with the study conducted in Jammu Province, India [30], which reported a higher prevalence in sheep (68.54%) compared to other species. In contrast to our study, host-wise prevalence of parasitic infection has shown that cattle have the greatest rate at 43.03 percent, followed by buffaloes at 40.8%, sheep at 29.4%, and goats at 21.4% [24]. The prevalence of GI nematodes is influenced by multifaceted factors, including economic conditions, farmer education, grazing management, and the types of anthelmintics used [31]. The observed increase in nematode infections in our study area may be attributed to compromised host immunity resulting from nutritional deficiencies [32], which enhances susceptibility to parasitic infections. Malnutrition exacerbates vulnerability to infection, and poor animal health diminishes resilience against infection-related symptoms [33].

Similar to our study, Win et al. [34] determined a higher occurrence of parasitic infections in females compared to males. Conversely, Abbas et al. [35] and Tiele et al. [25] reported a higher prevalence in males. A study by Felefel et al. [36] indicated higher infections in females (39.1%) than males (35%), though the difference was statistically insignificant. There may be variations in susceptibility to GI parasitic infections based on gender due to genetic predisposition and fluctuating susceptibility brought on by hormonal regulation [37]. Because of hormonal variations during pre-parturient and postpartum periods, it is frequently believed that females are more susceptible to parasitic infections. Testosterone’s reported immunosuppressive effects [38] might make males more susceptible to various infectious diseases [39].

Neighbouring regional studies suggested that the prevalence of parasitic diseases in ruminants could be influenced by age [40]. Our findings align with observations by Hassan et al. [41] and Tiele et al. [25], indicating that older ruminants are more vulnerable to GI parasites. However, some studies have reported a higher prevalence among young animals [3,42]. A study in Tangail, Bangladesh, by Rahman et al. [22] found that adult ruminants (65.11%) were more susceptible to parasitic infections than young ones (58.09%). Similarly, older ruminants exhibited higher susceptibility to nematodes, whereas coccidia infections were more prevalent in younger animals [43]. The higher prevalence in adults could result from reduced immunity with age, coupled with suboptimal animal management [44]. In our study, the higher prevalence of parasitic infections in adults might be attributed to keeping them for extended breeding periods or supplying inadequate feed to meet their high demands. Additionally, prolonged exposure to contaminated pastures could increase infection risk in older animals.

In the present study, a non-significant association between GI parasitic infections and months was observed. These findings are in contrast with Nayab et al.’s [45] study, which recorded a significantly lower prevalence in cows and buffaloes during March in Khyber Pakhtunkhwa, Pakistan. Abbas et al. [35] noted elevated prevalence during July and August in Punjab, Pakistan. Similarly, Rahman et al. [22] found significant seasonal disparities in Bangladesh, with rainy seasons having the highest prevalence (72.44%), followed by winter and summer (56.72% and 61.82%, respectively). The increased infection risk in our study area may be due to favorable environmental conditions for parasite development. These include suitable humidity and moisture, a temperature range (19–32 °C) conducive to larval maturation, and ample water for larval migration [46].

Geographic variations in parasite species prevalence can result from favorable environmental conditions, suitable intermediate hosts, and effective control methods. Various parasitic species, such as *Paramphistomum* spp., *Fasciola* spp., and *Moniezia* spp., were identified in the study area, consistent with previous reports [3,28,47]. Coprological examinations in Ethiopia revealed infection with strongyles (54.17%), *Strongyloides* (8.33%), *Trichuris* (3.13%), and mixed types (6.25%) [23]. Similarly, Rahman et al. [22] reported an overall prevalence of nematodes (52.11%), cestodes (2.11%), trematodes (36.62%), and protozoa (10.33%).

According to Raza et al. [21], mixed infections with nematodes and trematodes were discovered in 6.4% of all animals, mixed nematode–cestode infestations in 3.8% of all animals, and all three groups were found in 19.1% of animals. The poly-parasitism incidence of our study is consistent with research from other sites in Ethiopia [29] and Pakistan [16], which results in morbidity and productivity loss. According to Wang et al. [48], poly-parasitism reduces host immunity and makes animals more vulnerable to other infections. Numerous nematode species thrive in the surroundings of natural water reservoirs and may complete their life cycles without the aid of intermediary hosts [49]. Polyparasitism is common in this area due to a number of reasons. First off, the variety of habitats available to different parasite species increases the likelihood of numerous parasitic exposures. Second, the socioeconomic circumstances of many livestock owners frequently limit their capacity to execute thorough parasite control procedures, resulting in subpar management techniques and elevated infection susceptibility. Multiple parasite species can establish infections as a result of the lack of access to veterinary healthcare and diagnostic services, which also restricts early detection and treatment. The risk of polyparasitism is further increased by the ability of animal migration within and between regions to encourage the transmission of several parasites. These ecological, social, and healthcare-related elements work together to generate an environment that supports polyparasitism in ruminants.

The estimation of EPG or OPG serves as a measure of GI parasite burden. Marskole et al. [40] found EPG/OPG in the range of 201–300, consistent with our study. However, Rizwan et al. [16] reported a moderate burden of parasitic infections in most of the animals, followed in order by light and high parasitic burden. Overburdened parasites can compromise reproduction, growth rates, and animal productivity. A number of variables may have contributed to Faisalabad’s increased prevalence of low parasitic infection loads compared to moderate and high burdens. First, it is likely that the climatic and environmental factors in the area produce an environment that is less favorable for the survival and spread of parasites with severe burdens, leading to a higher proportion of animals with lower parasitic burdens. Second, more frequent and efficient deworming procedures could lessen the incidence of severe infections in Faisalabad. Additionally, the local ruminant population’s genetic makeup may make them more resilient to severe parasite infestations. These elements work together to increase the frequency of light parasite burdens in the area.

Iqbal et al. [50] in Lahore revealed organ-specific condemnation rates, with liver infection rates of 8.85% in sheep and 6.21% in goats. In Iran, Khedri et al. [51] estimated economic losses due to organ condemnation from parasites in animals. *Echinococcus granulosus*, *Fasciola* spp., and *Dicrocoelium dendriticum* were the main parasites causing condemnation. The total loss resulting from parasite-related condemnation during an eight-year period was calculated at USD 3,191,879. According to a study conducted by Opio et al. [52] in Uganda, each diseased animal suffered a financial loss of UGX 9900 (USD 2.67) due to liver impairment. In our study, parasitic infections caused 3.13% of the livers to be destroyed. This rate is consistent with research from Iran’s North Khorasan Province [53] but it is lower than that found in North Iran [54]. In Pakistan, most studies reported the condemnation of organs due to parasitic infection, but very few determined the estimated losses due to condemnation. An estimated economic loss because of parasitic infection-related organ contamination is provided in our study.

An important topic of concern is how gastrointestinal parasitic diseases in animals affect human health, particularly in the setting of neglected zoonotic diseases. The reports of zoonotic meta-cestodes (*Cysticercus bovis* and hydatid cyst), trematodes (*Dicrocoelium dendriticum*, *Eurytrema pancreaticum*, and *Fasciola*), and nematodes, (*Oesophagostomum*) entering the food chain in various parts of the world are of great concern from the standpoint of public health. Cystic echinococcosis and cysticercosis are possible outcomes of these parasitic infections in humans, along with diarrhoea, stunted growth, intellectual and cognitive impairment, and cysticercosis [16,51,55,56,57]. Ruhoollah et al. [58] reported a 13.58% *Fasciola* infection in ruminants in the district of Upper Dir in Pakistan’s Khyber Pakhtunkhwa Province. In a subsequent investigation by Khattak et al. [59], 4.53% *Fasciola* infection was reported in the Mardan district of Khyber Pakhtunkhwa. Abbas et al. [35] detected 12.48% *Fasciola* infection and 2.69% *Dicrocelium dendriticum* infection in the Pakistani area of Chakwal. Neglected zoonotic diseases place a significant burden on public health in affected areas, typically made worse by poor sanitation and restricted access to healthcare. For the purpose of creating comprehensive policies to reduce these hazards and enhance the general well-being of communities, addressing the relationship between animal parasite diseases and human health is essential.

In slaughterhouses, infected organs are often kept out of the market due to safety concerns. Instead, these diseased organs, regardless of their weight, are typically sold to local street vendors for a minimal sum of just Rs. 50 for small ruminants and Rs. 100 for large ruminants. However, the fate of these organs, or the portions of them purchased by local people, is often to be disposed of in rivers or along the roadside for animals as a form of charity (sadaqa). In our economic study, we have reasonably treated these organs as entirely condemned because they are effectively removed from the market and not consumed.

For ease of comparison and comparability among species, we used the same EPG ranges in the current investigation to classify low, medium, and high loads for various species. It enables simple data interpretation and simple comparisons between different parasites. This method might oversimplify the underlying complexity of parasite burdens and may be inaccurate in capturing species-specific changes. Another drawback of the current study is that although we examined the organs and collected the worms, we were unable to gather all of the worms from each diseased organ because of the butcher’s hurried efforts to go to the market. As a result, we were unable to more accurately describe the association between the load of adults and EPG.

## 5. Conclusions

The study carried out in the district of Faisalabad in Punjab, Pakistan, offers important new information on the prevalence and financial effects of parasitic diseases in the ruminant population. Goats showed the highest prevalence of parasitic infection (52.0%), followed by cattle (46.08%), buffalo (45.98%), and sheep (34.74%), and the overall frequency of parasitic infection was reported to be 44.79%. The prevalence of parasitic infection was considerably impacted by age and gender, with adult and female ruminants displaying greater prevalence rates. The month of slaughter, however, did not significantly correlate with parasite illness. Infections with a single parasite were more frequent than infections with numerous parasites in the majority of ruminants. Ten parasite species were identified by the investigation, with *Eimeria* oocysts being the most common. Other species were *Ostertagia, Trichostrongylus*, *Haemonchus*, *Trichuris*, Hydatid cysts, *Paramphistomum*, *Toxocara*, *Strongyloides*, and *Fasciola*. The prevalence of parasitic infection was highest in the intestine (29.43%), followed in order by the abomasum (18.49%), liver (6.25%), rumen (3.91%), and lungs (3.65%). In comparison, the economic losses due to condemnation were significantly higher in the rumen and lungs than in the liver. This resulted in a projected total annual economic loss of Pak. Rs. 133,731,400/- (equivalent to USD 466,939.23).

Indeed, the knowledge gained from our research illuminated previously unknown aspects of parasite diseases in ruminants in Faisalabad, Punjab, Pakistan. These results highlight the complex problems that the local livestock industry faces, both in terms of animal health and the financial effects of parasitic illnesses. Our study’s findings on the prevalence and impact of these infections serve as a sobering reminder that the full cost of parasitic diseases goes far beyond the manifestation of symptoms in animals. Along with the condemnation of infected organs, it includes decreased weight gain, decreased production, digestive disturbances, and lower reproductive effectiveness. Importantly, these unaccounted costs have a direct impact on the lives of countless people who depend on ruminants for food and money, which heightens the need for prompt action. The results of this study could be used to provide guidance on parasite prevention and control strategies to mitigate the costs to the livestock community.

## Figures and Tables

**Table 1:** Association of gastrointestinal parasitic infection with various factors in the ruminant population of district Faisalabad, Punjab, Pakistan.

Character	Variables	Examined	Positive	Prevalence	*X*^2^ Value	*p*-Value	95% Confidence Interval
Species	Cattle	102	47	46.1	6.103	0.107	45.9286, 46.1914
	Buffalo	87	40	46.0			45.9646, 46.0220
	Sheep	95	33	34.7			33.446, 35.514
	Goat	100	52	52.0			51.8899, 52.1768
Sex	Male	219	79	36.1	15.667	0.000	35.9292, 36.1841
	Female	165	93	56.4			55.706, 56.801
Age	Young	141	52	36.9	4.598	0.032	35.317, 37.870
	Adult	243	117	48.1			47.8936, 48.2731
Months	January	64	32	50.0	5.897	0.316	49.8899, 50.1768
	February	64	29	45.3			44.766, 45.641
	March	64	23	35.9			34.293, 36.934
	April	64	27	42.2			41.8606, 42.3328
	May	64	26	40.6			40.190, 41.297
	June	64	35	54.7			53.466, 55.460
* Parasitic burden	Light	172	80	46.5	22.047	0.000	45.960, 47.380
	Moderate	172	52	30.2			29.8327, 30.4539
	High	172	40	23.3			22.8151, 23.4915
Number of parasites	One parasite	172	112	65.1	154.692	0.000	64.9136, 65.2330
	Two parasites	172	46	26.7			26.4087, 27.2180
	Three parasites	172	14	8.1			7.9009, 8.2591

* identified only through faecal examination.

**Table 2:** Estimated annual economic losses due to the condemnation of organs due to parasitic infections in slaughtered ruminants of Faisalabad, Punjab, Pakistan.

Organ	Animal	Mean Number of Ruminants Slaughtered (a)	Mean Cost (b)	Prevalence (c)	Annual Loss = a × b × c (in Pak Rs.)	in USD
Liver	Large ruminants	10,680	1800	3.13	60,171,120	210,094.7
	Small ruminants	14,240	800	3.13	35,656,960	124,500.6
Lung	Large ruminants	10,680	450	1.3	6,247,800	21,814.9
	Small ruminants	14,240	200	2.34	6,664,320	23,269.3
Rumen	Large ruminants	10,680	700	2.6	19,437,600	67,868.7
	Small ruminants	14,240	300	1.3	5,553,600	19,391.1
Total					133,731,400	466,939.2

## Data Availability

All the data can be found in the main text.

## Supplementary Materials

The following supporting information can be downloaded at: https://www.mdpi.com/article/10.3390/pathogens12111291/s1, Table S1: Parasitic species present as singles, pairs, and combinations of three in ruminants of district Faisalabad.
