# Supplementary material for: Inflicting Significant Losses in Slaughtered Animals: Exposing the Hidden Effects of Parasitic Infections
Source: Pathogens. 2023 Oct 29;12(11):1291. doi: 10.3390/pathogens12111291 (PMC10674797; doi:10.3390/pathogens12111291)
Supplement: Supplementary file 1 [file pathogens-12-01291-s001.zip › pathogens-2677527-supplementary.pdf]

Supplementary Table S1. Parasitic species present as singles, pairs, and combinations of three in ruminants of district Faisalabad

**Parasitic Infection**	**Positive out of 172**	**Prevalence**
Eimeria oocyst	26	15.12
Trichuris	24	13.95
Haemonchus	22	12.79
Ostertagia	20	11.63
Toxocara	9	5.23
Strongyloides	6	3.49
Trichostrongylus	5	2.91
Hydatid cysts + *Trichuris*	7	4.07
Hydatid cysts + *Eimeria* oocyst	5	2.91
Paramphistomum + *Ostertagia*	4	2.33
Eimeria oocyst + *Trichostrongylus*	3	1.74
Hydatid cysts + *Ostertagia*	4	2.33
Fasciola + *Haemonchus*	2	1.16
Hydatid cysts + *Trichostrongylus*	3	1.74
Hydatid cysts + *Haemonchus*	3	1.74
Paramphistomum + *Trichuris*	3	1.74
Haemonchus + *Trichuris*	2	1.16
Paramphistomum + *Toxocara*	1	0.58
Fasciola + *Toxocara*	1	0.58
Paramphistomum + *Haemonchus*	1	0.58
Hydatid cysts + *Toxocara*	1	0.58
Hydatid cysts + *Strongyloides*	1	0.58
Fasciola + *Ostertagia*	1	0.58
Paramphistomum + Eimeria oocyst	1	0.58
Strongyloides + *Toxocara*	1	0.58
Paramphistomum + *Strongyloides*	1	0.58
Fasciola + *Trichostrongylus*	1	0.58
Hydatid cysts + *Haemonchus* + *Strongyloides*	2	1.16
Haemonchus + *Eimeria* oocyst + *Trichostrongylus*	2	1.16
Paramphistomum + *Eimeria* oocyst + *Toxocara*	1	0.58
Haemonchus + *Eimeria* oocyst + *Trichuris*	1	0.58
Paramphistomum + Hydatid cysts + *Ostertagia*	1	0.58
Fasciola + *Haemonchus* + *Strongyloides*	1	0.58
Paramphistomum + Hydatid cysts + *Haemonchus*	1	0.58
Hydatid cysts + *Ostertagia* + *Eimeria* oocyst	1	0.58
Hydatid cysts + *Haemonchus* + *Eimeria* oocyst	1	0.58
Hydatid cysts + *Eimeria* oocyst + *Trichuris*	1	0.58
Hydatid cysts + *Haemonchus* + *Trichostrongylus*	1	0.58
Paramphistomum + *Haemonchus* + *Eimeria* oocyst	1	0.58
